# Navigating the USPTO’s AI inventorship guidance in AI-driven drug discovery

**DOI:** 10.1093/jlb/lsaf014

**Published:** 2025-08-02

**Authors:** Joanna Wang

**Affiliations:** Insilico Medicine, 1000 Massachusetts Avenue, Suite 126, Cambridge, MA 02138, United States

**Keywords:** AI-driven innovation, artificial intelligence, drug discovery, inventorship guidance, patent, USPTO

## Abstract

In February 2024, the United States Patent and Trademark Office (USPTO) issued a notice, *Inventorship Guidance for AI-assisted Inventions* (‘Inventorship Guidance’), to clarify agency policy and the Office’s interpretation of inventorship requirements for patents that describe inventions made with the assistance of artificial intelligence (AI). From the perspective of an AI-driven drug discovery (AIDD)-focused business, the Inventorship Guidance offers potential benefits that include increased clarity in patent eligibility, facilitated collaboration between AI experts and drug discovery scientists, and incentivization for continued development of AI tools. However, there remain concerns with the application of the framework outlined in the Inventorship Guidance, such as the complex assessment of substantial human contributions in the real world, challenges in applying the Inventorship Guidance to collaborations and partnerships in the drug discovery field, and challenges in determining inventorship for AI tools versus specific drug innovations. To address these challenges, I propose recommendations for modifying the Inventorship Guidance for the AIDD industry, suggest best practices for inventorship documentation and processes, and advocate for continued partnership between the USPTO and the AIDD sector. By refining the existing framework and fostering ongoing dialog, I aim to promote a balanced approach that encourages AI-driven innovation while recognizing essential human contributions in drug discovery.

## I. INTRODUCTION

The pharmaceutical industry is undergoing a transformative shift driven by advances in artificial intelligence (AI) technology. AI-driven drug discovery (AIDD) approaches have the potential to accelerate nearly every phase of the drug discovery and development process, as evidenced by leading pharmaceutical companies and startups making significant investments in AI capabilities to gain a competitive edge.^1^ Traditional targeted drug development typically involves first identifying a disease-associated molecular target, ie a protein or other biomolecule that itself functions abnormally or regulates other abnormally functioning factors in the setting of disease. Targets can be identified through comparative gene expression analysis of disease tissues, gene knockdown or over-expression screens, chemical screens, or other experimental methods. With a target identified, researchers can then begin the process of finding a suitable drug to interact with and change the function of that target in the intended way. This painstaking task can involve screening vast libraries of drug molecules against cells *in vitro* or iteratively modifying the chemical structures of existing drugs known to target the same or similar factors. Any promising hits must also be assessed for their drug-like properties, such as bioavailability, pharmacokinetics, and toxicity, in pre-clinical or animal models before being used in clinical trials.

AI-based tools have been introduced at every step in this process.^2^ Major inroads have been made in early drug discovery, where AI-based platforms can identify novel drug targets previously overlooked or identified only through the integration of disparate data sources afforded by AI tools.^3^ AI-driven virtual screening methods can rapidly evaluate billions of compound structures to identify promising lead molecules, reducing the time and costs associated with traditional experimental screening,^4^ and generative chemistry AI platforms remove constraints of the known chemical space, allowing for the *de novo* design of a wider array of new drug-like molecules based on specified criteria, including absorption, distribution, metabolism, excretion and toxicity (ADMET) properties, instead of massively parallel chemical screens of known and pre-synthesized compounds.^5^ AI integration in drug development has truly become end-to-end with the recent development of algorithms to predict clinical trial outcomes as well.^6^ The first commercial company to publish experimental validation of AI-generated molecules was Insilico Medicine, which, since 2016, has leveraged advanced generative AI technology to accelerate every stage of drug discovery.^7^ Our AI systems have already identified novel disease-associated molecular targets and designed a novel small-molecule drug for the treatment of idiopathic pulmonary fibrosis (IPF), which is currently being evaluated in a Phase II clinical trial in the U.S.^8^ Entities developing these tools come in diverse commercialization models, from academic institutions publishing foundational computational methods and novel algorithms into the public domain, to biotechnology or pharmaceutical companies developing tools and proprietary molecules in-house, to AI companies offering use of their platforms in clinical research organization (‘CRO’) or software-as-a-service models. Individual entities have often implemented aspects of multiple models and frequently collaborate and partner with different types as well.

However, as these powerful AI systems become increasingly intertwined in the invention of new drugs and therapies, complex questions arise around inventorship and intellectual property rights. From training generative models on massive compound datasets to deploying AI for target identification and lead optimization, AI has become an integral tool at all levels and has truly become a driving force in drug innovation efforts. With AI playing such a pivotal role, providing clear guidance on inventorship standards for AI-assisted inventions is crucial for incentivizing continued investment and development in this field. The recent guidance from the United States Patent and Trademark Office (USPTO) on inventorship represents a significant step toward addressing these challenges.

## II. OVERVIEW OF USPTO’S AI INVENTORSHIP GUIDANCE

In February 2024, the USPTO issued long-awaited guidance on how inventorship should be determined for AI-assisted inventions, ie *Inventorship Guidance for AI-assisted Inventions* (‘Inventorship Guidance’). This Inventorship Guidance, like other USPTO guidance documents, does not carry the force of law, as reiterated in *In Re: Rudy* (2020)^9^. It serves to provide patent applicants and examiners with information and clarification of the Office’s obligations and interpretation of statutory law in its evaluation of patent applications, as provided in Title 35 of the United States Code and US case law. The Inventorship Guidance aims to provide transparency about how the USPTO will evaluate patent applications involving AI-assisted inventions while staying within the bounds of current legal framework. This approach allows the USPTO to adapt to technological changes without requiring legislative action, though some experts argue that more formal rulemaking may eventually be necessary to address the unique challenges of AI innovation.^10^ This Inventorship Guidance provides clarity in the wake of court decisions like *Thaler v. Vidal*,^11^ which confirmed that only natural persons can be listed as inventors on patents. Furthermore, it sought to strike a balance between ‘encouraging innovation involving AI while preserving moral and economic incentives for human inventors’.^12^ Despite not having the force of law, the Inventorship Guidance has become a *de facto* standard that significantly influences market practices, with patent examiners, AIDD companies, pharmaceutical firms, and healthcare entities regularly relying on it when evaluating patents, structuring commercial deals, and developing internal policies.

The core principles outlined by the USPTO revolve around analyzing the contributions of human inventors using the ‘Pannu factors’ established by case law:

1. Each inventor must contribute in some significant manner to the conception or reduction to practice of the invention,
2. Each inventor must make a contribution to the claimed invention that is not insignificant in quality, when that contribution is measured against the dimension of the full invention, and
3. Each inventor must do more than merely explain to the real inventors well-known concepts and/or the current state of the art.^13^

The Pannu factors, established in *Pannu v. Iolab Corp.*, 155 F.3d 1344 (Fed. Cir. 1998), have been the cornerstone of inventorship determination in U.S. patent law for over two decades.^14^ However, their application to AI-assisted inventions has been subject to debate. Some critics argue that the factors, particularly the emphasis on conception, may not adequately capture the iterative and collaborative nature of AI-driven innovation.^15^ Others contend that the requirement for ‘significant contribution’ becomes ambiguous when AI systems perform complex computational tasks that would be impossible for humans.^16^ Despite these criticisms, the USPTO has chosen to retain and adapt these factors for AI-assisted inventions rather than develop new criteria, aiming to maintain consistency with established patent law principles while addressing novel technological challenges.^17^

Undoubtedly, the Inventorship Guidance has significant implications for AI’s role in the drug discovery field. Importantly, the Inventorship Guidance stipulates that the use of an AI system by inventors does not automatically preclude them from qualifying as inventors, as long as their contributions meet the Pannu factors. In my view, it is essential to recognize the value of AI assistance in drug invention, as these platforms fundamentally drive the value of the entire pharmaceutical industry. Furthermore, the USPTO provided five guiding principles for applying the Pannu factors to AI-assisted inventions, which are particularly relevant for AIDD.

1. A natural person’s use of an AI system in creating an AI-assisted invention does not negate the person’s contributions as an inventor.
2. Merely recognizing a problem or having a general goal or research plan to pursue does not rise to the level of conception.
3. Reducing an invention to practice alone is not a significant contribution that rises to the level of inventorship.
4. A natural person who develops an essential building block from which the claimed invention is derived may be considered to have provided a significant contribution to the conception of the claimed invention even though the person was not present for or a participant in each activity that led to the conception of the claimed invention.
5. Maintaining ‘intellectual domination’ over an AI system does not, on its own, make a person an inventor of any inventions created through the use of the AI system.

For AIDD companies, the Inventorship Guidance represents a first attempt to map existing inventorship doctrine onto the AI paradigm.^18^ However, it leaves some ambiguities about how to assess significant human contributions when leveraging advanced AI technology across different stages of the drug development pipeline. Applying these principles in the complex and collaborative environment of AIDD may require more industry-specific examples and further clarification.

## III. POTENTIAL BENEFITS FOR AIDD COMPANIES

### III.A. Clarity and Consistency in Evaluating the Patentability of AI-Assisted Drug Inventions

One of the most significant benefits of this Inventorship Guidance is that it provides a clear inventorship framework for AI-assisted drug innovations, alleviating previous concerns about the patentability of AI-assisted inventions. While AI systems cannot be named as inventors on patents, the Inventorship Guidance clarifies that a natural person’s use of an AI system in creating an AI-assisted invention does not negate the person’s contributions as an inventor.^19^ Moreover, although there remain ambiguities in how to apply the Pannu Factors, particularly regarding the interpretation of ‘contribution in a significant manner’ and ‘contribution must not be insignificant in quality’, the Inventorship Guidance establishes the principle that the patentability of AI-assisted inventions should be evaluated and determined through a multi-dimensional approach. This is especially crucial for the AIDD industry, where the R&D process is lengthy and complex, and the involvement of AI technology further complicates or obscures the process. Therefore, the determination of patentability for AI-assisted drug-related inventions must involve a comprehensive consideration of multiple dimensions and factors.

Example 2 in the Inventorship Guidance illustrates this multi-dimensional approach by analyzing whether the two scientists, Marisa and Naz, made significant contributions to the invention. The analysis considers various aspects, including identifying inputs to the AI tool, identifying compound candidates outputted from the AI tool, characterizing the drug compounds, determining structural modifications of the generated compound, identifying methodology for preparing the compound, synthesizing the compound, and more. This comprehensive evaluation leads to the conclusion that the scientists made significant contributions to the conception or reduction to practice of the invention. Such a perspective and analysis are vital given the complex nature of the drug discovery process. In contrast to traditional contract research partnerships, AIDD companies involved in drug development projects are often more engaged in tool development and tailoring of AI tools to individual project needs, and their workflow involves a more multidisciplinary approach which requires deeper and more diverse expertise in application of their methods and interpretation of the resulting output. As the USPTO continues to refine its policies and guidelines for AI-assisted inventions, it is crucial that this multi-dimensional approach to evaluating AI-assisted inventions’ patentability be maintained and further developed. By considering the diverse range of factors involved in the inventive process, the USPTO can ensure that the unique challenges and contributions inherent to AIDD are properly recognized and assessed when determining patentability. In addition, the significance of Example 2 extends beyond its pharmaceutical context—it represents the USPTO’s attempt to provide practical guidance for an industry where AI’s role is rapidly expanding. The example illustrates how the USPTO intends to evaluate the complex interplay between human expertise and AI capabilities in modern drug discovery.^20^ Moreover, it sets important precedents for how contribution thresholds might be assessed in other AI-intensive fields.

### III.B. Facilitating Collaborations between AI Experts and Drug Discovery Scientists

Another significant benefit of the Inventorship Guidance is that it promotes and facilitates collaborations between AI experts and drug discovery scientists in the drug discovery process. The Inventorship Guidance acknowledges that AI-assisted inventions often involve multidisciplinary teams, and it provides clarity on how to attribute inventorship when such collaborations occur. Scenario 2 in Example 2 of the Inventorship Guidance illustrates this point effectively. Under this scenario, Marisa, a biotech researcher, collaborates with Raghu, an AI expert, to develop a novel drug compound using an AI system called Molecule Optimizer (‘MO’). The Inventorship Guidance explains that both Marisa and Raghu can be considered joint inventors of the AI-assisted invention because they made significant contributions to the development of MO and the resulting drug compound. Marisa’s contributions include identifying desirable properties for the drug compound, developing a scalar objective function based on these properties, and synthesizing and validating the drug compounds generated by MO. Raghu’s contributions involve developing MO, training it on relevant datasets, and fine-tuning the model based on Marisa’s feedback. By recognizing the significant contributions of both the biotech researcher and the AI expert, the Inventorship Guidance encourages and facilitates such collaborations in the real world. This clarity is crucial for the AIDD industry, where cross-disciplinary collaborations are becoming increasingly common and necessary. By providing a framework for attributing inventorship in these collaborations, the Inventorship Guidance reduces uncertainty and potential conflicts, fostering an environment where AI experts and drug discovery scientists can work together more effectively to develop innovative drug compounds.

### III.C. Incentivizing Continued Development of Powerful AI Tools for Drug Discovery

The Inventorship Guidance plays a crucial role in promoting innovation by incentivizing the continued development of powerful AI tools for drug discovery. By establishing that scientists do not lose inventorship rights when using AI tools in drug development, the Inventorship Guidance demonstrates respect for emerging technologies and encourages researchers to harness the power of AI in their work. This recognition is essential, as AI tools have the potential to revolutionize drug discovery by accelerating the process, reducing costs, and identifying novel drug candidates that may have been overlooked by traditional methods, which ultimately benefit all of humanity. For example, our company has demonstrated the unique power of AI by identifying potential inhibitors of the protein 3CLpro, a key target for treating COVID-19,^21^ and designing and synthesizing a novel drug candidate for IPF in only 46 days, compared to the typical timeline of several years.^22^ As industry insiders, we believe that encouraging the development and use of AI tools is critical for driving innovation in the pharmaceutical industry.

Moreover, the Inventorship Guidance ensures that the patent value of the AIDD sector is protected, which is crucial for attracting investment and fostering collaborations between AIDD companies and traditional pharmaceutical companies. Drugs and their associated patents in the drug discovery industry form the foundation of industry transactions, and collaborative drug development is crucial in this field. For example, the main types of transactions, such as license-out and collaborative development, are based on the underlying value of drug patents.^23^ Pharmaceutical companies generate revenue by holding patents related to their drugs, which grant them rights to exclusively sell the products during the patent term.^24^ In license-out transactions, the main consideration paid by the licensee is for the licensor’s patents related to their drug pipeline under development.^25^ In collaborative drug development, the parties also negotiate in detail the ownership of drug patents and the associated revenue sharing, which forms the cornerstone of their joint development investments.^26^ By providing a clear framework for attributing inventorship and protecting patent rights, the Inventorship Guidance creates a supportive environment for innovation in the AIDD industry. As the technology continues to advance, it is essential that the legal framework keeps pace and continues to incentivize the development of cutting-edge AI tools. The Inventorship Guidance is a significant step in this direction, promoting innovation and ensuring that the pharmaceutical industry can fully benefit from the transformative potential of AI in drug discovery.

## IV. CONCERNS AND CHALLENGES

### IV.A. Complexity in Assessing Significant Human Contributions in the Real World

The examples provided in the Inventorship Guidance are hypothetical and may not fully capture the complexity of the drug discovery process in the real world. In Example 2, the AI tool is assumed to be used solely for predicting drug compounds with high binding affinity to a mutated androgen receptor protein (AR). The scientists virtually screen existing compound datasets, and the AI tool’s function is simplified to only generate a numerical value for each drug compound, representing the binding affinity of the drug compound to the mutated AR. The objective of Example 2 is to illustrate how contributions to project conception, contemporaneous innovation, and experimental characterization and modification of compounds are sufficient for inventorship, whereas implementation of a pre-developed and pre-trained AI model using pre-existing data is not. However, in the real world, AI tools developed by companies like us are powered by various models and numerous algorithms utilizing generative AI technology.^27^ The process is not a simple input–output mechanism. Existing datasets are often used to train the models only during the AI tool development phase. When the AI tools are commercially launched, the underlying models are already well pre-trained, so users can simply insert target or compound information, set various screening criteria, and generate results through the AI tools without further training the platform on additional data. Moreover, when generating results, multiple variably weighted factors may be considered, and the inputs may be tested and optimized for various, sometimes oppositional, parameters that require scientists’ expertise and judgment in balancing.^28^ When AI tools generate candidate compound results, which could number in the hundreds, multiple rounds of setting adjustments and re-running the models may be necessary. Even after several iterations, human insights and expertise are still crucial for selecting compounds, rather than simply choosing the one with the highest numerical score.^29^ Similar to the example, further modification and synthesis of the selected compounds are essential steps in advancing the drug discovery process. Each of these steps requires the involvement of various and numerous contributors and subject matter experts, which reveals a more complicated process than the simplified version presented in the Inventorship Guidance example cases.

For example, as demonstrated in our INS018_055 study,^30^ the AIDD process may be deceptively intricate. The PandaOmics AI platform used for target identification integrates multiple data types, including gene expression, protein–protein interactions, and small molecule properties.^31^ It employs various AI approaches, such as natural language processing of scientific literature, network analysis, and matrix factorization to identify promising targets, eventually leading to identification of the kinase TNIK as a disease-associated molecular target for IPF. For compound generation, the Chemistry42 platform utilizes multiple generative models in parallel, exploring millions of potential structures.^32^ The process involves iterative optimization based on predicted binding affinity, drug-like properties, and synthetic accessibility. Inputs for this compound generation process included the crystal structure of the TNIK kinase domain and specific pharmacophore requirements, which are typically determined through collaboration and group discussions among multidisciplinary teams of chemists, biologists, and data scientists. The output was not a single compound, but rather a diverse set of candidate molecules that were then prioritized based on multiple criteria. This required significant human expertise to interpret the results and select the most promising candidates for synthesis and testing. It can thus be seen that significant human input, interpretation, and decision-making at multiple stages are necessary and essential. In addition, a substantial amount of subsequent experimentation in cells, experimental models, and animals is required to achieve even a basic level of target validation. This requires the additional cooperation of many scientists, veterinarians, medical doctors, and strategic leadership.

The involvement of numerous participants and the difficulty in quantifying their individual contributions make it challenging to assess ‘significant human contributions’ in practice. The boundaries between who does what in real-world scenarios are not as clear-cut as in the hypothetical examples.^33^ The complex nature of AIDD, involving multiple stakeholders, iterative processes, and the integration of human expertise at various stages, complicates the determination of inventorship in the real world compared with the example held in the Inventorship Guidance. This complexity highlights the need for a more nuanced approach to evaluating inventorship in AIDD, one that takes into account the collaborative nature of the process and the essential role of human judgment and expertise in guiding the development and application of AI tools.

### IV.B. Challenges and Ambiguities in Applying the Inventorship Guidance to Collaboration and Partnerships

While the Inventorship Guidance provides much-needed clarity on AI-assisted inventions in drug discovery, there remain challenges and ambiguities in applying the Inventorship Guidance to real-world collaborations and partnerships. In Example 2, the Inventorship Guidance makes it clear that an AI expert who merely operates an AI platform according to another person’s instructions, eg Raghu’s contribution, is not considered an inventor due to the insignificance of their contribution.^34^ However, in practice, collaborations are rarely as simple as following instructions. In many collaborations and partnerships, the steps involved are often jointly discussed and decided upon by both parties. Even in a service-based model, where AIDD companies typically assist pharmaceutical clients in identifying disease targets or designing compounds based on targets specified by the client, the party providing the service process does not simply input data into an AI platform following the client’s instructions and deliver the output to the other party. Service providers often contribute a significant amount of knowledge, expertise, and project-specific optimization and problem-solving in the process, taking the form of scientific discussions with the clients, review and analysis of generated results, result report drafting, scientific criteria review, etc.

In this framework, according to the Inventorship Guidance, as illustrated in Scenario 1, where the scientists were considered potential inventors, and in Scenario 2, where the AI expert was deemed a potential inventor, in a service model, if the drug discovery researchers or AI experts from AIDD companies acting as service providers satisfy the Pannu factors, they may be able to claim inventorship rights. However, this could pose challenges to the existing service model. Currently, relationships between AIDD companies and pharmaceutical clients operate under well-established frameworks with clearly defined deliverables and IP ownership structures. In these arrangements, IP rights for all deliverables typically belong exclusively to pharmaceutical clients, with AIDD companies rarely asserting claims to IP ownership. This market practice serves a crucial function: it ensures pharmaceutical companies maintain complete and unencumbered IP rights over drug compounds and assay results, enabling them to freely develop, commercialize, or license their drug pipeline without complications. Such clear IP ownership is fundamental to the value proposition in drug discovery deals, as potential partners, buyers, or licensees consider clean IP rights a critical factor in their due diligence and deal valuation.

However, if the Inventorship Guidance suggests that researchers or AI experts from AIDD service providers can claim inventor status upon meeting certain conditions, it could fundamentally alter these established dynamics. This shift might lead to protracted negotiations and potential disputes over IP rights to deliverables, with AIDD companies potentially seeking partial ownership or enhanced compensation for their inventive contributions. Such complications could significantly delay drug development timelines and create obstacles in the path to commercialization, ultimately impacting the efficiency of bringing new therapeutics to market. Additionally, unlike traditional service models where inventive contributions are typically predictable and easily contractually assigned, AI contributions may constitute core aspects of the drug discovery process itself.^35^ This could lead AIDD companies to demand patent rights to compounds or negotiate higher milestone payments and royalty shares. These complexities require the Inventorship Guidance to provide more clarity to ensure continued innovation and collaboration in the field while maintaining efficient pathways to drug development and commercialization.

One potential solution to ensure clear IP ownership in such services, based on the Inventorship Guidance, is to structure the service model in a way that the service provider operates the AI platform solely according to the client’s instructions, without making any additional contributions. However, this approach could potentially hinder the diversity of services and collaborations in drug discovery, as it may limit the service provider’s ability to offer valuable insights and expertise.^36^ Alternatively, both parties may need to reevaluate their expectations and establish clear terms regarding inventorship, IP ownership, payment structures, etc., which may transition a traditional service model to a strategic collaboration model to ensure a fair and mutually beneficial collaboration in light of the Inventorship Guidance. To address these challenges, it is crucial for collaborating parties to have open discussions and establish clear IP agreements that outline each party’s roles, responsibilities, and ownership rights. The Inventorship Guidance serves as a useful starting point, but it is ultimately up to the collaborating parties to navigate the complexities of their specific situation and ensure a fair and productive partnership.

### IV.C. Challenges in Determining Inventorship for AI Tools Versus Specific Drug Innovations

The Inventorship Guidance raises questions about handling inventorship for AI tools versus specific drug innovations, particularly in light of Principle 4. This principle states that in some situations, the natural person(s) who designs, builds, or trains an AI system in view of a specific problem to elicit a particular solution could be an inventor, where the designing, building, or training of the AI system is a significant contribution to the invention created with the AI system.^37^ The USPTO provides examples to illustrate this principle in Example 2. In Scenario 1, Lauren’s general training of a hypothetical AI model, Drug Target Interaction Predictor (DTIP), and general maintenance of the system is not a significant contribution to the conception of the invention of claim 1.^38^ The rationale is that these contributions were not made with a specific problem in mind or to elicit a particular type of output from DTIP to solve this problem.^39^ In contrast, under Scenario 2, the AI expert Raghu is deemed to contribute significantly to the conception of the claimed compound because he identified a problem with the output of established AI models (ie undesirable properties of previously synthesized AI-designed compounds), developed and trained the new AI system MO, which generated the novel particular drug compound that does not have the specific problem.^40^

It appears that for an AI developer to be considered an inventor of a compound, they need to bear a specific problem-solving concept in mind, develop the AI tool, and have it lead to problem-solving. However, this problem-solving logic may not always align with the reality of AIDD research and development processes. As previously discussed, AI tools often consist of multiple underlying models and not every model is designed with the goal of producing a specific type of molecule or a better one.^41^ The logic behind AI development and drug discovery is different - AI model training focuses more on improving prediction accuracy and efficiency than producing a specific molecule that treats a disease. Some AI models may aim to screen dataset quality, improve prediction accuracy, refine generated results, or other purposes unrelated to drug discovery, which is not the same concept as designing a molecule with better efficacy and desirable ADMET in drug discovery.

Moreover, the purpose of training AI models is usually more general, such as training the model to consider not only potential efficacy but also tolerant toxicity when generating molecules. However, this training purpose is not typically intended to generate a specific class of molecules or treat a particular disease. Therefore, it is unclear whether this type of problem-solving constitutes a contribution recognized by Principle 4. If it does, could relevant developers of these models be listed as inventors of the AI models (if patentable) or compound or both where it may conflict with the interests of scientists who substantially contributed to the compound invention? If not, following the idea that solving a specific problem is necessary to be listed as an inventor, either no AI developers would be eligible to be listed as inventors in practice as their contributions are ‘general’ in the real world, which would fail to encourage AI innovation in the pharmaceutical field, or only AI developers who develop AI to solve so-called ‘specific problems’ could be listed while others cannot. In that case, the incentive structure for developing the underlying models for AI tools may be disrupted, as these models are crucial for the success of AIDD but may not be considered as solving specific problems. This challenge is particularly significant because AI models in drug discovery differ from conventional research tools like statistical packages or general-purpose software. Generative AI systems in drug discovery actively contribute novel intellectual content through their ability to learn, generate, and optimize new chemical entities.^42^ The AI models themselves embody significant innovation in how they navigate vast chemical spaces and identify previously unexplored therapeutic opportunities.^43^ As noted by industry experts, these systems do not merely compute—they create and invent through sophisticated algorithms that combine chemical, biological, and therapeutic knowledge in unprecedented ways.^44^

On the other hand, AI technology itself is relatively difficult to protect with patents,^45^ which already poses challenges for incentivizing innovation in this field. This difficulty stems from both the abstract nature of AI algorithms and their rapid evolution. When AI developers create sophisticated models that enable novel drug discoveries, their intellectual contribution extends beyond providing a mere tool—they are enabling new paths of invention that would be impossible through traditional methods.^46^ The Inventorship Guidance, while aiming to clarify inventorship in AI-assisted inventions, may inadvertently create a disincentive for AI innovation if it fails to recognize these fundamental contributions. This could particularly impact the development of sophisticated AI models that, while not targeting specific therapeutic problems, create the essential foundation for drug discovery breakthroughs. This creates a challenging paradox: the very innovations that make AIDD possible may go unrecognized and unrewarded under current patent frameworks. Therefore, we believe that more detailed rules and guidance are necessary to address these regulatory challenges and ensure that the patent system effectively promotes and protects AI innovation in the pharmaceutical industry.

## V. RECOMMENDATIONS

### V.A. Proposed Modifications to IP Guidance for the AIDD Industry

The importance of properly attributing inventorship in AIDD cannot be overstated, as it impacts not only the validity of patents but also the incentives for innovation in this rapidly evolving field. The Inventorship Guidance is a significant step forward, but it needs refinement mainly in three key areas to better address the unique challenges of AIDD.

First, I propose that the USPTO expand its guidance with more detailed, industry-specific examples that better reflect the complex reality of AIDD processes. The Inventorship Guidance should provide more detailed criteria for evaluating contributions in iterative drug discovery processes. Current examples present oversimplified, linear scenarios that don’t reflect the reality of modern AIDD, where multiple rounds of AI-assisted discovery and optimization occur in parallel. The guidance should explicitly address how to evaluate cumulative contributions across these iterations. Also, it needs to clarify the role of compound synthesis in determining inventorship. The Inventorship Guidance may overemphasize the importance of compound synthesis itself, which, in the context of AIDD, is often a standard follow-up step rather than a significant intellectual contribution. AI-based computational tools have even been developed to themselves compute molecular synthesis routes to validate the feasibility of output drug molecules.^47^^,^^48^ Even so, AI platforms have not yet reached the level of autonomy where they can produce the exact compounds with good efficacy and low toxicity without human intervention. In practice, compound synthesis is frequently performed by dedicated experimentalists or contract research organizations, rather than the primary researchers or companies who actually discover the compound. Attributing inventorship based on synthesis alone could lead to misallocation of credit and potential disputes over IP ownership. Instead, the focus should be on identifying those who conceptualized the compound and made significant intellectual contributions to its discovery.

Furthermore, the interpretation of ‘specific problem-solving’ in Principle 4 requires revision for AIDD applications. While the current guidance emphasizes solutions to specific therapeutic problems, it may consider recognizing valuable contributions from developing general-purpose AI capabilities that enable drug discovery breakthroughs. This is particularly crucial given that many fundamental AI innovations in drug discovery arise from improving general capabilities rather than solving specific therapeutic challenges. In the meantime, the Inventorship Guidance’s use of the term ‘significant in quality’ when evaluating inventive contributions, as described in the Pannu factors, requires further clarification in the context of AIDD. Example 2 focuses primarily on binding affinity and selective binding to mutated AR. However, in the pharmaceutical industry, the quality of a contribution could be interpreted more broadly. Does ‘quality’ refer to drug efficacy, reduction of side effects, or advancements in the drug discovery process? I propose that the USPTO provide more nuanced guidance on how to interpret ‘significant in quality’ specifically for AIDD inventions. For instance, whether a contribution that leads to a compound with marginally better efficacy but significantly fewer side effects could be considered more ‘significant in quality’ than one that only improves efficacy slightly? Similarly, could a contribution that dramatically reduces the time or cost of discovering a drug compound, even if the resulting compound is only incrementally better, be considered significant? The future guidance may acknowledge that in AIDD, the quality of a contribution might be measured not just by the end product, but also by improvements in the efficiency, speed, or cost-effectiveness of the discovery process.

Moreover, the USPTO should further clarify and provide examples that capture the iterative and collaborative nature of AIDD. The guidance should establish clearer frameworks for evaluating inventorship in complex collaborative scenarios. This should include specific criteria for assessing contributions when multiple teams use various AI tools at different stages of drug discovery, particularly in cases where improvements to AI systems occur alongside drug development. Examples also should illustrate how to assess contributions in scenarios involving multiple teams or organizations, such as collaborations between AIDD companies and pharmaceutical companies. This would help stakeholders navigate the complex landscape of joint inventorship in AIDD projects.

### V.B. Best Practices for Inventorship Documentation and Processes

To address the challenges of determining inventorship in the AIDD field, we recommend implementing robust documentation and communication practices that align with and expand upon the disclosure requirements outlined in the Inventorship Guidance. The Inventorship Guidance emphasizes the importance of the duty of disclosure under 37 CFR 1.56(c) and the duty of reasonable inquiry under 37 CFR 11.18(b). Building on these requirements, companies should establish clear guidelines for recording human contributions at each stage of the discovery process. This aligns with the USPTO’s emphasis on significant human contributions and helps ensure compliance with the duty of disclosure. We have implemented a documentation process where detailed logs or memos should be maintained to capture key decisions, insights, and contributions made by team members throughout the AIDD process. These records should be sufficiently comprehensive to demonstrate how each named inventor has satisfied the Pannu factors, particularly in terms of their contribution to the conception of the invention. In line with the Inventorship Guidance’s call for a ‘reasonable inquiry’ into inventorship, companies should implement regular review processes involving both scientific and legal teams. These reviews should assess potential inventorship claims and ensure that all material information regarding inventorship is promptly disclosed to the USPTO. This practice not only fulfills the duty of reasonable inquiry but also helps preempt potential disputes over inventorship. Moreover, in light of the Inventorship Guidance’s acknowledgment of the complexity of AI-assisted inventions, companies should consider developing AI-specific inventorship policies. These policies should provide clear guidelines on how to assess and document the contributions of both human inventors and AI systems, ensuring that the company can readily demonstrate compliance with the USPTO’s inventorship requirements.

In the meantime, transparency should extend beyond patent filing to client discussions, ensuring that inventorship issues are addressed early in collaborations. This aligns with the Inventorship Guidance’s emphasis on the importance of clear communication regarding inventorship. I anticipate an increased need for deep-dive due diligence, comprehensive disclosure requirements, and specific representations and warranties related to inventorship in various agreements in the drug discovery industry. These measures can help prevent misunderstandings and ensure that all parties are aware of their responsibilities regarding inventorship disclosure. Also, companies should develop clear communication channels between AI developers, drug discovery scientists, and legal teams. This interdisciplinary approach can facilitate ongoing discussions about inventorship and help identify any potential issues early in the invention process. It can also assist in gathering the necessary information to respond effectively to any requests for information under 37 CFR 1.105, as mentioned in the Inventorship Guidance.

### V.C. Continued Partnership Between USPTO and AIDD Sector

The rapidly evolving nature of the AIDD industry necessitates an ongoing dialog between the USPTO and key stakeholders in the AIDD field. I recommend establishing regular forums for exchange, such as annual workshops or roundtables, where industry leaders, researchers, and patent officials can discuss emerging challenges and best practices in AIDD patenting. Also, the USPTO may consider creating a formal mechanism for the AIDD sector to provide feedback on the practical application of the Inventorship Guidance. This iterative approach would allow for continuous improvement of the guidelines, ensuring they remain relevant and effective. Furthermore, the formation of an industry working group could help develop sector-specific best practices for inventorship determination and documentation in AIDD. Additionally, collaboration between the USPTO and the AIDD sector could extend to the development of AIDD-specific patent examination guidelines. These guidelines should address the unique challenges of AIDD inventions, taking into account the complex interplay between human expertise and AI capabilities. Additionally, investing in specialized training programs for patent examiners and industry players would enhance their understanding of AI technologies and their application in drug discovery, leading to more informed and consistent patent examinations.

By implementing these recommendations, the USPTO can foster a more robust, clear, and equitable framework for patenting AI-assisted drug inventions. This approach would not only promote innovation but also ensure proper recognition of human contributions in this rapidly advancing field, ultimately benefiting the entire pharmaceutical industry and, by extension, global public health.

## VI. CONCLUSION

In conclusion, the Inventorship Guidance represents a significant step toward clarifying the complex issues surrounding inventorship and patent protection in the AIDD sector. AI has the potential to revolutionize drug discovery by enabling researchers to navigate the vast chemical space more efficiently and effectively, ultimately leading to the discovery of new therapies for patients in need.^49^ As the pharmaceutical industry increasingly embraces AI tools to accelerate the discovery and development of new medicines, it is crucial to have a clear and equitable framework for recognizing and rewarding the contributions of both human inventors and AI systems. The Inventorship Guidance acknowledges the critical role of AI in expediting the drug discovery process, reducing costs, and identifying novel drug candidates that may have been inaccessible with traditional methods. By providing a pathway for AI-assisted inventions to be patented, the Inventorship Guidance encourages the continued development and adoption of cutting-edge AI technologies in the pharmaceutical industry.

However, as discussed here, the Inventorship Guidance also raises important questions and challenges that need to be addressed to ensure a balanced and equitable approach to inventorship and IP protection in AIDD. The pharmaceutical industry, AI developers, and policymakers must work together to refine and adapt the existing framework to promote AI-driven innovation while also recognizing the essential contributions of human inventors.^50^ To achieve this goal, I recommend that stakeholders engage in ongoing dialog and collaboration to develop inventorship standards and policies that are transparent, consistent, and adaptable to the rapidly evolving landscape of AI in drug discovery. This may involve refining the Inventorship Guidance to provide more specific criteria for evaluating inventorship in AI-assisted inventions, as well as establishing best practices for documenting and attributing contributions in multi-disciplinary collaborations.^51^

Ultimately, the success of AIDD will depend on the ability of the patent system to strike a delicate balance between promoting innovation and protecting the rights of inventors. By embracing the opportunities presented by AI while also addressing the unique challenges it poses, we can create a legal framework that supports the development of groundbreaking new therapies and ensures that the benefits of AIDD are broadly accessible to patients worldwide.

## Footnotes

1 Lalitkumar K. Vora et al., *Artificial intelligence in pharmaceutical technology and drug delivery design*, 15 Pharmaceutics 1916 (2023).

2 Jessica Vamathevan et al., *Applications of machine learning in drug discovery and development*, 18 Nat. Rev. Drug  Discov. 463–477 (2019).

3 Frank W. Pun et al., *AI-powered therapeutic target discovery*, 44 Tips. 561–572 (2023).

4 Hongming Chen et al., *The rise of deep learning in drug discovery*, 23 Drug  Discov. Today. 1241–1250 (2018).

5 Yan A. Ivanenkov et al., *Chemistry42: An AI-driven platform for molecular design and optimization*, 63 J. Chem. Inf. Model. 695–701 (2023).

6 Alex Aliper et al., *Prediction of Clinical Trials Outcomes Based on Target Choice and Clinical Trial Design with Multi-Modal Artificial Intelligence*, 114 Clin. Pharm. Therap. 972–980 (2023).

7 Alex Zhavoronkov et al., *Deep learning enables rapid identification of potent DDR1 kinase inhibitors*, 37 Nat. Biotechnol. 1038–1040 (2019).

8 Feng Ren et al., *A small-molecule TNIK inhibitor targets fibrosis in preclinical and clinical models*, 43 Nat. Biotechnol. 63–75 (2025).

9 In re Rudy, 956 F.3d 1379, 1383 (2020).

10 Erin Hanson & Sahra Nizipli, *USPTO provides guidance on the patentability of AI-assisted inventions*, https://www.whitecase.com/insight-alert/uspto-provides-guidance-patentability-ai-assisted-inventions (accessed Jan. 26, 2025).

11 Thaler v. Vidal, 43 F.4th 1207, 1213 (Fed. Cir. 2022).

12 Inventorship Guidance, Section III. B (2024).

13 Inventorship Guidance, Section IV. A (2024).

14 Todd M. Hopfinger & Emily S. Tkac, *Navigating Inventorship of AI-Assisted Inventions: USPTO’s Guidance and Implications*, https://www.sternekessler.com/news-insights/publications/navigating-inventorship-of-ai-assisted-inventions-usptos-guidance-and-implications/ (accessed Jan. 26, 2025).

15 Micheal H. Rubin, P. Anthony Sammi & Jamie D. Underwood, *USPTO Releases Guidance on AI and Inventorship*, https://www.lw.com/admin/upload/SiteAttachments/USPTO-Releases-Guidance-on-AI-and-Inventorship.pdf (accessed Jan. 24, 2025).

16 Douglas R. Nemec & Laura M. Rann, *AI and Patent Law: Balancing Innovation and Inventorship*, https://www.skadden.com/insights/publications/2023/04/quarterly-insights/ai-and-patent-law (accessed Jan. 26, 205).

17 Hanson, *supra* note 11.

18 Inventorship Guidance, Section V., B(I) (2024).

19 Inventorship Guidance, Section IV.B, principle 1 (2024).

20 Hanson, *supra* note 11.

21 Jing Sun, et al., *A novel, covalent broad-spectrum inhibitor targeting human coronavirus M^pro^*, 16 Nat. Comm. 4546 (2025). 

22 Zhavoronkov, *supra* note 8.

23 Thomas Klueter, L. Felipe Monteiro & Denise R. Dunlap, *Standard vs. partnership-embedded licensing: Attention and the relationship between licensing and product innovations*, 46 Res. Policy. 1629–1643 (2017).

24 Ashish Kumar Kakkar, *Patent cliff mitigation strategies: giving new life to blockbusters*, 25 Expert  Opin. Ther. Pat. 1353–1359 (2015).

25 Steven Simoens, *Pricing and reimbursement of orphan drugs: the need for more transparency*, 6 Orphanet J. Rare  Dis. 42 (2011).

26 Sean Nicholson, Patricia M. Danzon & Jeffrey McCullough, *Biotech-pharmaceutical alliances as a signal of asset and firm quality*, 78 J. Bus. 1433–1464 (2005).

27 Zhavoronkov, *supra* note 8.

28 Kit-Kay Mak & Mallikarjuna Rao Pichika, *Artificial intelligence in drug development: present status and future prospects*, 24 Drug  Discov. Today. 773–780 (2019).

29 Debleena Paul et al., *Artificial intelligence in drug discovery and development*, 26 Drug  Discov. Today. 80–93 (2021).

30 Ren, *supra* note 9.

31 Petrina Kamya et al., *PandaOmics: An AI-driven platform for therapeutic target and biomarker discovery*, 64 J. Chem. Inf. Model. 3961–3969 (2024).

32 Ivanenkov, *supra* note 6.

33 Martin Kretschmer, Bartolomeo Meletti & Luis H. Porangaba, *Artificial intelligence and intellectual property: copyright and patents - a response by the CREATe Centre to the UK Intellectual Property Office’s open consultation*, 17 J. Intellect. Prop. Law. 321–326 (2022).

34 Inventorship Guidance, Example 2 (2024).

35 Nic Fleming, *How artificial intelligence is changing drug discovery*, 557 Nat. 55–57 (2018).

36 Inventorship Guidance, Section II (2024).

37 Inventorship Guidance, Section IV.B, principle 4 (2024).

38 Inventorship Guidance, Example 2, Scenario 1 (2024).

39 Inventorship Guidance, Example 2, Scenario 1 (2024).

40 Inventorship Guidance, Example 2, Scenario 2 (2024).

41 Zhavoronkov, *supra* note 8.

42 Neil Savage, *Tapping into the drug discovery potential of AI*, Nat. Biopharm. Deal. B37–B39 (2021).

43 Alexandre Blanco-González et al., *The Role of AI in Drug Discovery: Challenges, Opportunities, and Strategies*, 16 Pharmaceutics 891 (2023).

44 Dolores R. Serrano et al., *Artificial Intelligence (AI) Applications in Drug Discovery and Drug Delivery: Revolutionizing Personalized Medicine*, 16 Pharmaceutics 1328 (2024).

45 Mark A. Lemley & Bryan Casey, *Remedies for robots*, 86 Univ. Chic. Law  Rev. 1311–1396 (2019).

46 Fleming, *supra* note 36.

47 M. Parrot, H. Tajmouati, V.B.R. da Silva et al. *Integrating synthetic accessibility with AI-based generative drug design*. J Cheminform, 15, 83 (2023).

48 L. Long, R. Li, J. Zhang. *Artificial Intelligence in Retrosynthesis Prediction and its Applications in Medicinal Chemistry*. 68 J. Med. Chem. 2333–2355 (2025), https://doi.org/10.1021/acs.jmedchem.4c02749

49 Michael Liebman, *The role of artificial intelligence in drug discovery and development*, 44 J. Chem. Int. 16–19 (2022).

50 Information Technology Industry Council, *ITI Policy Principles for Enabling Transparency of AI Systems*, https://www.itic.org/documents/artificial-intelligence/ITIsPolicyPrinciplesforEnablingTransparencyofAISystems2022.pdf (accessed Oct. 16, 2024).

51 Inventorship Guidance, Section I, background (2024).

